# Stakeholder perspectives on the use of patient-reported outcome measures in colorectal cancer survivorship care in general practice: qualitative study using interviews

**DOI:** 10.1007/s11136-025-04116-5

**Published:** 2026-01-21

**Authors:** Bora Kim, Marguerite Tracy, Cheri Ostroff, Janani Mahadeva, Julie Marker, Kate White, Simon Willcock, Claudia Rutherford

**Affiliations:** 1 The Daffodil Centre, The University of Sydney, and Cancer Council NSW, Sydney, NSW 2006 Australia; 2 Cancer Care Research Unit, Sydney Local Health District, Sydney, NSW 2006 Australia; 3 Faculty of Medicine and Health, General Practice Clinical School, The University of Sydney, Sydney, NSW 2006 Australia; 4 Susan Wakil School of Nursing and Midwifery, The University of Sydney, Sydney, NSW 2006 Australia; 5 Faculty of Medicine, Health and Human Sciences, Macquarie Medical School, Macquarie University, Sydney, NSW 2109 Australia; 6 Consumer representative, Adelaide, SA 5000 Australia; 7 Cancer Voices SA, Adelaide, SA 5068 Australia; 8 Centre for Workplace Excellence, UniSA, Adelaide, SA 5001 Australia

**Keywords:** Colorectal cancer, Patient-reported outcomes, Primary health care, General practitioner, Sequelae, Cancer survivorship

## Abstract

**Purpose:**

To explore perspectives of colorectal cancer (CRC) survivors and general practitioners (GPs) regarding the potential use of Patient-Reported Outcome Measures (PROMs) in survivorship care.

**Methods:**

We conducted a qualitative study with semi-structured interviews with CRC survivors and GPs to explore their opinions on the potential utility, feasibility, and preferred method of PROMs utilization in their care/clinical practice. Thematic analysis was conducted using a qualitative descriptive approach.

**Results:**

Three themes emerged from 13 CRC survivor interviews:(1) potential of PROMs as tools for revealing and monitoring hidden CRC sequelae, (2) benefits and drawbacks to be weighed when using PROMs in CRC survivorship care, and (3) practical strategies to enhance the utility of PROMs in CRC survivorship care. Two themes emerged from four GP interviews:(1) current uses of PROM in general practice may indicate potential applications in CRC survivorship care, and (2) implementation of PROMs in CRC survivorship care needs to consider resource-limited general practice environments. CRC survivors had positive views about using PROMs in their care, believing they could be a valuable tool to enhance monitoring of treatment sequelae/and communication with their GP. GPs also acknowledged the potential utility but emphasized that for CRC-specific PROMs to be implemented, they first needed to demonstrate tangible improvements to the current way that CRC treatment sequelae were monitored and managed, considering already resource-limited Australian general practice settings.

**Conclusions:**

Future research assessing the feasibility of integrating CRC-specific PROMs into general practice settings requires a particular focus on the resource implications within these settings

**Supplementary Information:**

The online version contains supplementary material available at 10.1007/s11136-025-04116-5.

## Abstract (unstructured)

Those who have completed treatment for colorectal cancer (CRC) often experience various post-treatment effects and concerns, such as fecal incontinence, sexual dysfunction, and anxiety about recurrence. While there is considerable knowledge about the benefits and feasibility of using patient-reported outcome measures (PROMs) to improve the quality-of-life for individuals with cancer within cancer services, there is limited understanding regarding their feasibility in general practice settings. This study explored the perspectives of CRC survivors and general practitioners (GPs) regarding the potential use of PROMs in survivorship care.

Most CRC survivors expressed a positive view towards using PROMs in their care, believing that these tools could assist in recognizing treatment impacts and enhance communication with their GP. GPs emphasized the necessity for CRC-specific PROMs to clearly demonstrate tangible improvement in the way that CRC impacts were monitored and managed, with careful evaluation of resource implications, considering already resource-limited Australian general practice settings.

This study highlights the importance of recognizing the unique general practice setting and that knowledge gained from cancer services regarding the use of PROMs may not directly apply to this context. Future research should examine how cancer-specific PROMs, like those for CRC, can fit into the current workflow of general practitioners.

**Supplementary Information:**

The online version contains supplementary material available at 10.1007/s11136-025-04116-5.

## Introduction

Advances in effective diagnosis and treatment of Colorectal Cancer (CRC) have improved survival rates, resulting in a significant rise in the number of individuals living with and beyond CRC (referred to as CRC survivors). In 2023, Australia is estimated to have 15,400 new cases of colorectal cancer, ranking it as the fourth most diagnosed cancer [1]. The number of CRC survivors is expected to reach 239,000 by 2040 [2].

The standard treatment for CRC includes different combinations of surgery, chemotherapy and radiotherapy. The sequelae of these treatments can continue for many years after treatment ends or be lifelong [3], affecting CRC survivors’ health-related quality of life (HRQL). Mobility issues were experienced by as many as 65% of CRC survivors three years after primary treatment, while 40% experienced pain and discomfort [4]. Additionally, within the same period, as high as 83% reported incontinence, and a considerable number of patients faced challenges with their sexual and urinary functions [4]. Bowel-related symptoms such as fecal incontinence and urgency, can worsen over time in a subset of patients beyond one-year post-treatment [5]. Psychological sequelae and practical challenges are also common such as emotional difficulties, difficulty socializing, financial burden and return-to-work challenges [3].

Current models of post-treatment care for CRC survivors in Australia are inadequate to address these immediate/late-occurring effects of treatment, some having to self-manage persistent issues through trial and error and also relying on information from online sources which may not be evidence-based [6]. There is a need for a shift in focus from simply detecting cancer recurrence to the management of a range of survivorship issues holistically to ensure that this growing number of CRC cancer survivors achieve optimal health and HRQL outcomes [7].

Primary care can be a means to meeting the increasing demand for care outside acute services, stabilize healthcare costs and provide care close to home [8]. To accommodate the growing number of cancer survivors in the community, GPs’ expertise in chronic and complex illness management becomes critical within cancer survivorship care. The GP-led survivorship care model has been shown to be a viable alternative to the specialist-led model, offering easy access and lower costs while achieving similar levels of patient HRQL [9]. Despite indications that GP-led models are a viable alternative to specialist-led models [9], providing high-quality care will depend on adequately assessing the wide array of potential sequelae, which can often be challenging within time-constrained clinics in these settings [10].

Adequately monitoring patient-reported outcomes (PROs) using patient-reported outcome measures (PROMs) might have a role in general practice by providing a means of screening for treatment sequelae that warrant intervention and enabling appropriate referral/clinical pathways for managing the identified issues to improve patient satisfaction and HRQL, and possibly extend survival [3, 11]. Given that symptoms and functioning impairments from cancer and its treatment can be experienced long after active treatment has been completed, it is important that PRO assessments continue beyond the acute treatment period.

Some may perceive the treatment sequelae as part of their ‘new normal’ and may not consider them as something that could be intervened [12]. Under-reporting of CRC treatment sequelae has been previously documented, with many symptoms and functional difficulties experienced not being discussed with their GPs [13]. This communication gap was especially prominent for topics of a sensitive nature (e.g., fecal incontinence, sexual dysfunction) and psychosocial issues (e.g., anxiety, isolation) [13]. This highlights missed opportunities, as there is a wide range of management options available within the Australian health system. Under-managed cancer treatment sequelae can not only diminish an individual’s quality of life but also have a negative societal impact through reduced productivity [14].

Using PROMs to promptly communicate about various issues and concerns has the potential to alter the quality of their post-treatment care. However, most PROM use in the clinical practice setting has been at the cancer treatment stage, and few studies have investigated the use of PROMs in general practice post-cancer treatment [15]. This study aims to explore the perspectives of CRC survivors and GPs, regarding the potential use of PROMs in routine general practice care for monitoring and managing CRC treatment sequelae. It specifically examines their opinions on the potential utility, feasibility, and preferred method of utilization.

## Materials and methods

### Study design

We conducted a descriptive exploratory study using semi-structured qualitative interviews with CRC survivors and GPs.

### Participants

CRC survivors aged ≥ 18 years who had completed their primary treatment regime for a CRC, and GPs with experience of caring for CRC survivors in Australia, who spoke English, and were able to give written informed consent to take part were eligible for the study.

#### ***Recruitment***

Multiple recruitment methods were used, including electronic advertisements through professional primary care (*n* = 8) and consumer (*n* = 7) organizations across Australia, email invitations to investigators’ collegial networks, and social media promotion. Social media promotion was conducted through Twitter and Facebook. The Twitter promotion was carried out using the organizational account to facilitate the distribution of the study invitation through existing networks. Relevant Facebook groups were identified using keywords such as, but not limited to: ‘cancer’, ‘colorectal cancer’, ‘colon cancer’, ‘rectal cancer’, ‘cancer survivorship’, and ‘Australia General Practice’. Facebook groups in which the study investigators were members were also included. A total of 11 Facebook groups were identified (7 = consumer groups, 4 = GP groups). Where group administrators granted permission, the study invitation was posted for distribution to their members (7 = consumer groups, 3 = GP groups). Members in the included Facebook groups ranged from 707 to 50,000. Most organizations and social media groups shared the study invitation more than once to assist in meeting the recruitment target.

An electronic advertisement contained the participant information sheet and a link to express interest for an interview. A snowball approach was also used, where participants were encouraged to share the study information with others who may be eligible. All study participants provided electronic consent. Ethics approval was granted by the University of Sydney Human Research Ethics Committee, under Project No: 2020/851 (approved 03/02/2021). Initial ethics approval was obtained with no financial compensation offered for interview participation. However, as recruitment took place during the COVID-19 pandemic, when general practices were experiencing increased service demands, recruiting GP participants proved challenging. As a result, financial compensation was later offered to GP interview participants to compensate for their practice time. Ethics approval was subsequently obtained for the financial remuneration.

### Data collection

This was a sub-study of a larger program of research aimed at understanding current CRC survivorship care in general practice and the role of the GP in coordinating post-treatment care in Australia [6]. Between March and October 2021, BK conducted individual semi-structured interviews with patient and GP participants. The interviewer (BK) had no prior caring or working relationship with the participants. Of those participating in this study, the researcher asked if they would be willing to provide their perspectives on the use of PROMs for CRC survivorship care in the GP setting. Those who agreed to proceed to the second part of the interview provided data for the current study. The interview schedule was developed by consumers (CO, JM) and GP investigators (JM, MT), and PROM expert (CR) with multiple iterations, focusing on exploring participants’ opinions on the potential utility, feasibility, and preferred method of PROMs utilization in their care/clinical practice (Supplementary A). Mock interviews were also conducted with the consumer (CO, JM) and GP (JM) investigators with hypothetical scenarios to evaluate the flow of the interview questions and to explore possible probing questions. Interviews were conducted via telephone or Zoom, depending on participants’ preferences. For the CRC survivor interviews, recruitment ceased when data saturation was reached; consistent themes emerged from the interviews, and contrasting opinions were also identified to capture diverse perspectives. For the GP interviews, recruitment was closed when all available recruitment strategies had been exhausted, and the project reached the grant project timeline.

### Data analysis

We transcribed audio recordings verbatim and conducted thematic analysis using a qualitative descriptive approach, which seeks to describe qualitative data by focusing on explicitly expressed participants’ accounts [16]. A qualitative descriptive approach was chosen as the study aimed to understand participants’ thoughts and opinions rather than exploring lived experiences that may require examination of implicit meanings behind their narratives. Data from the survivor and GP groups were interpreted and presented separately due to their fundamentally different roles and contexts in cancer care.

The thematic analysis was conducted to identify themes inductively by two researchers (BK, CR). Specifically, the first reviewer (BK) developed the initial coding schema, and the second reviewer (CR) confirmed the codes by reviewing the transcripts. BK and CR developed preliminary and final themes through iterations of coding, examining data, and discussion. Discrepancies were resolved through discussions.

BK is a registered nurse with a background in oncology and hematology nursing, and CR has expertise in PROM development and implementation. Both researchers’ professional backgrounds and knowledge were used to inform the interpretation of the data, with deliberate efforts made to minimize bias in data interpretation by engaging in reflexive discussions during the thematic analysis.

## Findings

Thirteen CRC survivor participants (Table 1) and four GP participants (Table 2) shared their opinions on the potential use of PROMs for monitoring and managing CRC treatment sequelae in general practice settings. Most CRC survivor participants were female (*n* = 9), from metropolitan areas (*n* = 9), and had a diagnosis of colon cancer (*n* = 10). All GP participants were female and based in metropolitan areas, with three having over 20 years of experience as general practitioners. Interview lengths ranged from 17 to 34 min, with a mean of 21 min. Our study team did not observe any noticeable differences in the depth of the qualitative data between those who participated via Zoom or telephone.

**Table 1:** Colorectal cancer participant characteristics

**Patient ID**	**Sex**	**Geographic location**	**Year diagnosed**	**Age at diagnosis**	**Cancer location**	**Cancer stage**	**Treatment received**
P1	M	Metropolitan	2016	20–29	Colon	2	Surgery/CT
P2	M	Regional	2005	60–69	Rectum	2	Surgery/CT
P3	F	Regional	2020	50–59	Colon	3	Surgery/CT
P4	F	Metropolitan	2018	30–39	Colon	3	Surgery/CT
P5	M	Metropolitan	2015	40–49	Colon	3	Surgery/CT
P6	F	Metropolitan	2017	50–59	Colon	3	Surgery
P7	F	Rural	2020	50–59	Rectum	2	Surgery
P8	F	Metropolitan	2011	50–59	Colon	2	Surgery
P9	F	Metropolitan	2018	30–39	Colon	3B	Surgery/CT
P10	M	Metropolitan	2019	50–59	Rectum	2–3	Surgery
P11	F	Metropolitan	2017	30–39	Colon	2	Surgery/CT
P12	F	Metropolitan	2019	50–59	Colon	3B	Surgery/CT
P13	F	Rural	2019	60–69	Colon	2	Surgery/CT

CT chemotherapy

### Findings from CRC survivor interviews

Majority of CRC survivor participants saw the potential benefits of integrating a standard set of PROMs into their routine care within general practice settings. While some participants expressed concerns about the potential time burden of using PROMs, many viewed them as tools that can allow for more efficient communication with their GPs by helping them better prepare for consultations and make it easier to raise sensitive issues. Most participants preferred self-completed electronic PROMs over clinician-assisted completion and emphasized the importance of incorporating mechanisms that facilitated tangible supportive care measures, such as providing evidence-based resources and connecting them with relevant services.

**Table 2:** General practitioner participant characteristics

Patient ID	Sex	Geographic location	Years of practice	Age	Working status	Average number of colorectal cancer patients seen annually
GP1	F	Metropolitan	30	60–69	Part-time	2
GP2	F	Metropolitan	4	30–39	Part-time	3
GP3	F	Metropolitan	40	60–69	Part-time	5
GP4	F	Metropolitan	20	50–59	Part-time	10

#### **Potential of PROMs as tools for revealing and monitoring hidden CRC sequelae**

Several participants described ways in which PROMs can benefit their care after CRC treatment, with a specific emphasis on their role in effectively identifying and communicating treatment sequelae that might otherwise be overlooked. For example, one participant in her 30s with busy day-to-day commitments thought that self-completed PROM scores could help her become more self-aware of certain problems that she ‘*may be hiding*’ and enable her to think, ‘*maybe I need to do something about it’.* Two other participants also stressed that some ongoing treatment-related issues could be left unresolved, and suggested using PROMs could help identify these issues and support individuals in finding management options.

Sometimes we don’t necessarily think that things might be a problem. A lot of things become normal for us…we forget that that’s actually not like that. There are some things we shouldn’t be dealing with all the time … there might be actual solutions to them rather than us just trying to cope with it and move through it every day. (P1 Man 20–29 years, 5 years post-treatment, Metropolitan area).

Another described that intermittent issues were often forgotten during GP appointments and having PROMs could prevent the oversight of those issues.

You often think, ‘OK, I’ll remember to ask this,’ but then you walk out and you forget…it was one of those side effects that passed, but at the time it worried you. Maybe in a paper form [of PROMs]…would be helpful… (P12 Woman 50–59 years, 2 years post-treatment, Metropolitan area).

Another potential benefit of routine PROMs was described as having a tool to monitor their progress with their GP, for example as one described, being able to see whether ‘*doing better than what they reported a year ago*’. Additionally, another participant thought that the ability to compare their health status to general CRC survivors might provide reassurance knowing that other people also experienced similar issues.

This would be a prime example of what would have been really useful to me to know, that everyone else was feeling just like that too. Using that sort of questionnaire tool might also help people know that others are experiencing the same problems and where they are at. (P8 Woman 50–59 years, 10 years post-treatment, Metropolitan area).

#### **Benefits and drawbacks to be weighed when using PROMs in CRC survivorship care**

Some participants raised concerns about the language used in PROMs for individuals with varied health literacy, and accuracy of the reported sequalae, as well as time constraints and possible health survey burden on both CRC survivors and GPs.

GP might not get time to read it [PROMs remotely completed by the patient]…Less paper the better. There’s gotta be something that’s really simple to work off, and not everyone is literate and educated, so the simplest things are the better for everyone. (P6 Woman 50–59 years, 4 years post-treatment, Metropolitan area).

However, other participants shared a contrasting view that PROMs could enable more efficient care with their GPs. They believed that completing PROMs prior to appointments could save valuable consultation time, as it would allow CRC survivors to organize their thoughts before the consultation and prioritize their issues for discussions. One in particular stated:

Really like the idea. If a survey [PROMs] was sent out prior to every checkup, it potentially saves time in the GP appointment with questions asked in advance so things for discussion are prioritized. Chemo-brain fog is a real thing, and a comprehensive list of questions of side effects could also act as a bit of a memory jog for any ‘niggling’ side effects the patient wants to bring up (P9 Woman 30–39 years, 3 years post-treatment, Metropolitan area).

Two participants expressed concern about the limited utility of PROMs as CRC survivors may under-report their issues by sharing their perception that “*patients are often hesitant to report*,* and many tend towards under-reporting*.” However, others thought that PROMs could help better elicit concerns and communicate with their GP. For example, one participant shared how she found certain sensitive issues, such as concerns with mental health and sexual functioning, difficult to raise with her GP. She described it as “*a little bit awkward talking about it*,” despite having a good relationship with her GP. She believed that PROMs could provide an opportunity for discussing these issues by taking “*a bit of stigma out*” as they would just be one of the other domains discussed as part of overall survivorship care. Another participant also thought that a standard set of PROMs could be a useful tool that help structure GP consultations for a comprehensive assessment of CRC treatment sequelae.

I absolutely think that that would be very good [using PROMs]…If you are a GP, maybe for the first time, talking about all of those sorts of things [for example, a GP could say], ‘We just wanna have a quick check over all of these areas to make sure that we haven’t missed anything that should be discussed. (P4 Woman 30–39 years, 3 years post-treatment, Metropolitan area).

#### **Practical strategies to enhance the utility of PROMs in CRC survivorship care**

Participants discussed strategies to ensure the feasibility of using PROMs in routine GP care for the ongoing monitoring of CRC treatment sequelae. The format of PROMs was considered important, including concise questions using simple language understandable to the general public and relevant to CRC survivors’ experiences. For example, one participant stressed that the PROM questions should be kept fairly broad and concise. Otherwise, they thought that it could overwhelm patients by stating “*anything that’s too onerous and there are lots of questions… people will just go*,* oh my goodness*,* this is crazy*.” Another participant suggested that the questions could remain relevant to the individual by making them customized based on the answers given so that “*it goes into areas that the person is concerned about in particular*.” The type of language used in PROMs needed to consider the health literacy of the general public. For example, one participant provided an example that “*incontinence*” would commonly be interpreted as urinary incontinence; therefore, suggested that lay terminology be considered.

There were mixed preferences for the mode of PROM delivery. Some preferred in-person clinician-assisted PROM completion for an individualized approach. For instance, one participant preferred their GP to ask the questions so that they could expand on why they gave a particular score by being able to say *“Well*,* at the moment*,* it’s two*,* but last week it might have been 10. Then these are the reasons why”.* They believed the consultation would be more personable and productive. However, more participants preferred to complete PROMs electronically in their own time for convenience.

I think it’s something that we can do…with less pressure [self-completed electronic PROMs]. Something that’s like an app or an e-mail, or you can just click your forms and add a couple of comments like as you go… with kids, I am busy. If it is fairly short then I can do it easily. I can even do it like you know, regularly every three months, six months, whatever, as a check-in type thing. (P9 Woman 30–39 years, 3 years post-treatment, Metropolitan area).

Several participants also emphasized the importance of having mechanisms for addressing concerning PROM scores to enhance their utility. In particular, one participant highlighted the importance of timely intervention in response to concerning PROM scores, stating that they are “*only effective when captured and actioned in a timely manner*.” Mechanisms for actioning concerning PROM scores were deemed important, such as linking with support services. For example, a participant living in a rural area described how identifying services to manage her urinary continence took a lengthy period, and having such a mechanism would have been helpful in her case. Another patient living in a rural area also thought that having “*something to go to straight away would be brilliant*.” Another participant also stressed that the provision of personalized evidence-based resources based on PROM scores would also be helpful, given the vast amount of online resources with varying credibility.

The other aspect I consider is how technology enables the delivery of personalized content. In my experience…it was challenging to find quality information. The first thing you are told when diagnosed with cancer is not to Google it, but if you don’t Google it, you are restricting your information, and when you do Google it, there is so much out there that is not evidence-based. (P11 Woman 30–39 years, 4 years post-treatment, Metropolitan area).

### Findings from GP interviews

GPs also acknowledged the potential utility of CRC-specific PROMs and expressed that their implementation may be feasible, given the existing use of PROMs in managing some non-cancer conditions. However, within the current resource-limited environment of Australian general practice, participants emphasized that for CRC-specific PROMs to be implemented, they must first demonstrate added clinical benefits and greater efficiency in managing CRC sequelae compared to the current methods of generic comprehensive patient assessment.

#### **Current uses of PROM in general practice may indicate potential applications in CRC survivorship care**

All GPs described that PROs were already collected and used in their day-to-day clinical assessments of patients, as one described ‘*general practice is kind of using patient reported outcomes because they* (patients) *come in and we take the history…we ask them all about how they’re doing’.* Some PROMs were already incorporated into structured care plans for certain conditions such as pain and mental health, but none existed that focused on cancer-specific care. Some general practices also used pre-populated care plan templates imported into their practice software to facilitate the collection and use of PROs data in patient care and referral to support services, as one described,

We offer mental health plans and have a comprehensive computer system for creating referral care plans. (GP4 50–59 years, 20 years practice experience, Metropolitan area).

Given that PROMs were already used in other non-cancer areas with structured assessment templates incorporated into the practice software, two GPs felt that a similar workflow could be adapted for CRC survivorship care to ensure cancer-specific issues were not overlooked.

There may be issues that patients do not mention, so having this system [structured PROM collection] could work. It would be best to have a template with the questions uploaded to our computer programs. Patients can complete the questionnaire either in the practice or at home. (GP3 60–69 years, 40 years practice experience, Metropolitan area).

#### **Implementation of PROMs in CRC survivorship care needs to consider resource limited general practice environments**

There were mixed views on the potential utility of CRC-specific PROMs for survivorship care. Two perceived benefits of having mechanisms such as “[having] *a list of symptoms that are completed by the patient with the link to the relevant resources*” for efficient consultation, as it would eliminate GPs’ efforts to seek the most relevant and evidence-based resources and services. Despite seeing the benefits, one GP stressed that the routine uptake of CRC-specific PROMs would only occur if such system added value without extending clinic time.

If there was a way of tracking how they were going and whatever that prompted them to follow up with us about the issue at a certain level then that could work, but. As I said, whatever it is with general practice, it won’t be taken up if it’s not saving or time neutral. (GP2 30–39 years, 4 years practice experience, Metropolitan area).

Other GPs questioned the utility of using cancer-specific PROMs in their existing workflow. One participant mentioned that they already ‘*need to take a thorough history anyway’*, during GP consultations, so one must carefully evaluate whether adding cancer-specific PROMs would bring tangible benefits. Another participant emphasized that implementing PROMs specific to CRC survivors would require demonstration of additional value ‘*beyond what is already done’*, such as linking with external services and allied health, before it can be considered for routine clinical use.

## Discussion

This study explored the perspectives of CRC survivors and GPs regarding the potential use of PROMs in routine general practice care for monitoring and managing CRC treatment sequelae, with particular focus on their potential utility, feasibility, and preferred methods of utilization. In general, CRC survivor participants expressed a favorable view towards integrating PROMs into their routine survivorship care, with the majority believing it would be beneficial for them. Consistent with a study that reported CRC patients’ perceived benefits of PROMs within specialist cancer nurse follow-up care [17], our study participants also described PROMs would provide an opportunity to identify issues that might have otherwise been overlooked or forgotten and prioritize issues that are most important to them to receive personalized support. Additionally, they believed that PROMs could enable easier discussion of sensitive health concerns with their GPs; concerns that are often underreported and under-managed and therefore remain an unmet need [3]. A survey of GPs on their views about the use of PROMs in non-cancer-specific care also identified PROMs as effective clinical management tools that facilitate shared decision-making with patients [18].

With respect to the feasibility of using PROMs in routine CRC survivorship care, some CRC survivor participants raised concerns about the burden associated with PROM completion for both patients and GPs, consistent with previous research [18], while others believed that the use of PROMs could enable more efficient GP consultations. Efficient consultations and time savings have been noted as benefits of using PROMs by primary care providers in the context of mental health care [19], and strategies can also be employed to ensure its effective integration in clinical care. For instance, clinician training to provide an opportunity to familiarize themselves with the PROMs aided efficient use [20]. The evolution of PROMs methodology also offers opportunities for adaptive electronic PROMs to customize questions based on patient responses, allowing collation of the most essential and relevant list for each individual without having to complete lengthy pre-determined static questionnaires [21]. This aligns with the preference for electronic PROMs expressed by survivor participants in the current study.

Due to the small sample size, drawing conclusions about GP’s preferences regarding the use of PROMs in CRC survivorship care was not possible. Nevertheless, this study highlighted that PROMs were already being used in some general practices for managing other non-cancer conditions and were embedded in structured assessment forms. Some GPs believed that using cancer-specific PROMs may be feasible in CRC survivorship care for comprehensive monitoring of treatment sequelae, but questioned the necessity of such a tool given that they felt they already had means to comprehensively assess their patients’ needs using generic assessment tools. Importantly, one stressed that PROM uptake would depend on the added practical value without extending clinic time, which aligns with the view of GPs in another study in the context of non-cancer-specific care [18]. Not all primary care providers view cancer survivors as a meaningfully distinct patient group that warrants a different care approach [22].

Nonetheless, given many post-CRC sequelae are underreported and consequently can go unmanaged within the community [3] and the lack of clarity exists among GPs about how to screen for cancer treatment sequelae [23], there is a need for a more effective and sustainable care approach for this patient group. Research into the use of CRC-specific PROMs as a potential solution needs further exploration, with a particular focus on their utility and feasibility within general practice settings. Future research would benefit from exploring what motivates GPs to use condition-specific PROMs, whether that be familiarity with the form, perceived benefits, workplace culture, or established workflows that allow efficient clinical care. The implementation of communication tools in Australian general practice settings is heavily influenced by their time implications [10], an important factor that must be considered when assessing the feasibility of using PROMs in general practice. The process of implementing PROMs is just as important as the content and performance of the PROM tools. Much work is needed to understand whether the integration of CRC-specific PROMs can be efficiently done within the routine workflow of general practices.

Much is known about the perspectives of individuals with cancer and clinicians on the use of PROMs in routine cancer care, focusing on their perceived utility, feasibility, and barriers/enablers to implementation [17, 24–27]. However, the evidence is mainly limited to cancer or palliative service settings, with primary care receiving less attention. This study contributes to the current literature by exploring the perspectives of CRC survivors and GPs regarding the potential use of PROMs for CRC survivorship care in the general practice setting. It adds important value given there is a growing recognition of the role of general practice in cancer survivorship [28], especially in CRC care in the context of an increasing population of CRC survivors [29] and their long-term need for managing treatment sequelae [3].

### **Limitations**

Given that recruitment in this study involved various consumer organizations and social media promotion, patients with lower health literacy and limited access to technology may be underrepresented in this study. Poor research participation among individuals with lower health literacy has also been reported elsewhere, which poses challenges for equitable access to research opportunities and the generalizability of findings [30]. In future research, disparities in research participation by level of health and/or digital literacy can be partly mitigated through diverse recruitment strategies, for example, by disseminating study invitations both online and in person (e.g., through health clinics), and by conducting research in partnership with consumer representatives who can ensure that study materials, including promotional flyers, are written in plain language appropriate for the general public [31].

The qualitative findings from GPs were reported with a small sample due to recruitment barriers during the COVID-19 pandemic, a period when general practices faced high service demands. Therefore, the application of the findings should be limited to drawing initial insights into the possible uses of and barriers to CRC-specific PROMs in the general practice setting and informing future studies with a larger and more diverse sample of GPs.

## Conclusion

This study presents the perspectives of CRC survivors and GPs regarding the potential use of PROMs in CRC survivorship care for monitoring and managing treatment sequelae in general practice settings, focusing on their potential utility, feasibility, and preferred method of use. CRC survivors generally expressed a favorable view of using PROMs in their post-treatment care, as it could aid in identifying treatment sequelae and facilitate communication with their GPs. While some GPs acknowledged the potential benefits, they emphasized that CRC-specific PROMs need to add tangible value to their existing workflow, especially in resource-limited Australian general practice settings. This underscores the importance of recognizing the uniqueness of the general practice setting and that knowledge gained from cancer services regarding the use of PROMs may not directly apply to this context. Future research efforts are needed to understand the compatibility of cancer-specific PROMs within the existing workflow of general practices.

## Supplementary Information

Below is the link to the electronic supplementary material.

Supplementary Material 1
