# Supplementary material for: Stakeholder perspectives on the use of patient-reported outcome measures in colorectal cancer survivorship care in general practice: qualitative study using interviews
Source: Qual Life Res. 2026 Jan 21;35(2):48. doi: 10.1007/s11136-025-04116-5 (PMC12823719; doi:10.1007/s11136-025-04116-5)
Supplement: Supplementary file 1 — Supplementary Material 1 [file 11136_2025_4116_MOESM1_ESM.docx]

***Supplementary***

Supplementary A. Interview schedules.

| **Colorectal cancer survivor interview:**  You may be aware that some health settings use structured questionnaires to monitor commonly experienced symptoms and other patient issues. Do you feel it would be useful to have a system that asked you about any symptoms that you experienced and provided support based on this report? For example, let’s say you experience severe pain, the results might prompt a referral for you from your GP to a pain specialist or you might be sent links to self-management options to try; and if you had no or very mild pain you might receive written feedback letting you know that you are tracking well but if the pain worsens to contact your GP.   1. Do you feel that such a system might be useful for patients after finishing treatment for CRC in terms of managing issues as they arise? 2. Do you feel that such a system could help you communicate about your symptoms and issues with your GP or other healthcare provider? 3. [If answered YES to Q1 or 2]: How do you see it being used? How often do you think it should be completed? In what format? 4. [If answered NO to Q1 or 2]: What is it about such a system that you think wouldn’t be helpful or useful in your care with a general practitioner?   **General practitioner interview:**  You may be aware that some health settings use structured patient-reported outcome questionnaires (sometimes called PROMs) to detect and monitor commonly experienced symptoms and other patient issues. As part of such interventions, information from the questionnaires is fed back to a designated healthcare professional who is responsible for acting on reported issues. In primary care, this might be the general practitioner or general practice nurse. For example, a patient may score their pain on a scale from 1 to 10 and those who experience severe pain may be referred to a pain specialist; someone who has moderate pain may be sent links to self-management options to try; and someone who has no or very mild pain may receive written feedback to let them know they are tracking well but if their pain worsens to contact their healthcare provider.   1. Do you think that such an intervention might be useful in primary care to care for colorectal cancer survivors? 2. If yes, what would be the expected benefits and uses? 3. If no, why do you feel this would not be useful? 4. If no, is there a different mechanism that would be a useful way to monitor symptoms and functional impairments in primary care? |
| --- |
